# Framework for developing explainable artificial intelligence models for neglected tropical disease diagnosis in low-resource settings

**DOI:** 10.3389/fdgth.2026.1900500

**Published:** 2026-08-10

**Authors:** David Chinaecherem Innocent, Rejoicing Chijindum Innocent, Increase Praise Innocent

**Affiliations:** 1 Machine Learning and Computational Research Cluster, Centicini Research Lab, Centicini, Abuja, FCT, Nigeria; 2 Department of Epidemiology, School of Public Health, University of Port Harcourt, Port Harcourt, Rivers State, Nigeria; 3 Pharmaceutical and Biomedical Research Cluster, Centicini Research Lab, Centicini, Abuja, FCT, Nigeria

**Keywords:** deep learning, diagnostic systems, explainable artificial intelligence, global health, low-resource settings, machine learning, neglected tropical diseases

## Abstract

**Background:**

Neglected tropical diseases (NTDs) continue to affect more than one billion people globally, disproportionately impacting populations living in low-resource settings characterized by limited diagnostic infrastructure, shortages of trained healthcare personnel, and restricted access to specialist services. Recent advances in artificial intelligence (AI), particularly deep learning and computer vision, have demonstrated significant potential for improving disease detection through the analysis of clinical images and microscopy data. However, despite encouraging diagnostic performance, many AI systems remain difficult to interpret, creating barriers to clinical trust, adoption, regulatory acceptance, and sustainable implementation in endemic regions.

**Main body:**

This narrative review examines the current landscape of AI applications in NTD diagnosis and critically evaluates the role of explainable artificial intelligence (XAI) in addressing challenges associated with transparency and trustworthiness. Evidence from studies involving malaria, schistosomiasis, soil-transmitted helminth infections, leishmaniasis, and skin-related NTDs demonstrates the growing capacity of AI to support diagnostic decision-making in resource-constrained environments. Nevertheless, persistent challenges related to limited datasets, poor data quality, algorithmic bias, model drift, infrastructure constraints, and ethical governance continue to impede translation into routine healthcare practice. Existing explainability approaches, including Gradient-weighted Class Activation Mapping (Grad-CAM), heatmaps, Shapley Additive Explanations (SHAP), Local Interpretable Model-Agnostic Explanations (LIME), and attention mechanisms, were reviewed to assess their relevance for NTD diagnostic systems.

**Framework development:**

Drawing upon current evidence in explainable AI, digital health implementation, and global health systems research, a seven-stage framework is proposed comprising: (1) problem definition, (2) data acquisition, (3) model development, (4) explainability layer integration, (5) clinical validation, (6) deployment in low-resource settings, and (7) continuous learning and monitoring. The framework embeds explainability throughout the AI development lifecycle to enhance transparency, accountability, clinical relevance, and equity.

**Conclusions:**

Artificial intelligence has considerable potential to improve NTD diagnosis in low-resource settings, but successful adoption depends on trust, transparency, and usability. The proposed framework provides a structured pathway for developing explainable AI systems that are technically robust, clinically meaningful, ethically responsible, and implementable within resource-constrained health systems, thereby supporting future NTD control and elimination efforts.

## Introduction

Neglected tropical diseases (NTDs) comprise a heterogeneous group of communicable diseases that predominantly affect populations living in conditions of poverty, inadequate sanitation, limited healthcare access, and fragile health systems. The World Health Organization (WHO) currently recognizes 21 diseases and disease groups as NTDs, including leishmaniasis, schistosomiasis, lymphatic filariasis, onchocerciasis, trachoma, and soil-transmitted helminth infections. Despite being largely preventable and, in many cases, treatable, NTDs continue to impose a substantial burden on global health, particularly across sub-Saharan Africa, South Asia, and parts of Latin America. Recent WHO estimates indicate that approximately 1.5 billion people remain in need of interventions against NTDs worldwide, highlighting the persistent inequities that characterize the distribution of these diseases (1). Beyond their direct clinical consequences, NTDs contribute significantly to disability, stigma, educational disruption, reduced productivity, and entrenched cycles of poverty, making them both a health and development challenge (2, 3).

Recognizing this burden, the WHO launched the NTD Road Map 2021–2030, which outlines ambitious targets including a 90% reduction in the number of people requiring NTD interventions, a 75% reduction in disability-adjusted life years (DALYs) attributable to NTDs, and the elimination of at least one NTD in 100 countries by 2030 (3, 4). However, achieving these targets remains contingent upon strengthening diagnostic capacity, which continues to represent one of the most neglected components of NTD control programmes. Many NTDs present with overlapping clinical manifestations, are prevalent in geographically remote regions, and often require specialized laboratory expertise for accurate diagnosis. Conventional diagnostic approaches such as microscopy, molecular assays, and serological testing may be unavailable, unaffordable, or difficult to sustain in resource-constrained settings. Consequently, delayed or inaccurate diagnosis remains common, contributing to ongoing transmission, inappropriate treatment, and poor health outcomes. Furthermore, the global shortage of trained healthcare professionals, particularly laboratory scientists and specialist clinicians in endemic regions, further exacerbates diagnostic delays and undermines disease surveillance efforts (3).

Recent advances in artificial intelligence (AI), particularly machine learning, deep learning, and computer vision, have generated considerable interest as potential solutions to diagnostic challenges in low-resource environments. Artificial intelligence refers to computational systems capable of performing tasks that traditionally require human intelligence, while machine learning enables algorithms to learn patterns from data and improve performance without explicit programming. Deep learning, a subset of machine learning based on artificial neural networks, has demonstrated remarkable performance in image classification, object detection, and pattern recognition tasks. Within healthcare, these capabilities have facilitated the development of AI-assisted diagnostic systems capable of analysing skin lesions, microscopic images, radiological scans, and other clinical data with levels of accuracy approaching or, in some cases, exceeding human experts (5). For NTDs specifically, deep learning models have shown promise in the detection of malaria parasites, schistosome eggs, leishmaniasis lesions, and other disease manifestations using smartphone-based imaging and digital microscopy platforms, offering opportunities to expand access to diagnostic support in underserved communities (6, 7).

Despite these advances, a critical challenge continues to limit the translation of AI innovations into routine clinical practice: the lack of explainability. Many state-of-the-art AI systems operate as “black-box” models, producing predictions without providing understandable explanations regarding how decisions are reached. While high predictive accuracy is important, healthcare decision-making requires transparency, accountability, and trust. Clinicians are often reluctant to rely on diagnostic recommendations when the reasoning behind predictions remains unclear, particularly in high-stakes settings where incorrect decisions may have serious consequences for patients (8, 9). Moreover, concerns regarding algorithmic bias, fairness, reproducibility, and regulatory compliance have intensified calls for the adoption of Explainable Artificial Intelligence (XAI), which seeks to make AI decision-making processes more transparent and interpretable (10, 11). While explainability has received increasing attention in fields such as radiology, oncology, and cardiovascular medicine, its application to NTD diagnosis remains fragmented and poorly conceptualized. Existing studies predominantly focus on model performance metrics, with limited attention to how explainability can be systematically incorporated into AI development pipelines designed for low-resource settings.

This gap is particularly important because the success of AI-enabled NTD diagnostics will depend not only on predictive performance but also on their acceptability, trustworthiness, usability, and alignment with local healthcare realities. A framework that integrates explainability from the earliest stages of model development may therefore provide a more sustainable pathway toward equitable and scalable implementation. Accordingly, this review aims to propose a practical framework for developing explainable artificial intelligence models for neglected tropical disease diagnosis in low-resource settings.

## Methods

This study employed a narrative review methodology combined with a conceptual framework development approach to synthesize current evidence on XAI for NTD diagnosis and to propose a practical framework for implementation in low-resource settings. Narrative reviews are particularly appropriate when addressing emerging and multidisciplinary fields where evidence originates from diverse methodological traditions and where the objective extends beyond evidence aggregation to theory and framework development (12, 13). The review was informed by established guidance for narrative and integrative reviews to ensure transparency, rigor, and reproducibility throughout the synthesis process (14, 15).

Relevant literature was identified through structured searches of PubMed, Scopus, Web of Science, IEEE Xplore, and Google Scholar. Search terms included combinations of “Artificial Intelligence”, “Machine Learning”, “Deep Learning”, “Explainable Artificial Intelligence”, “XAI”, “Computer Vision”, “Neglected Tropical Diseases”, “Diagnosis”, “Digital Health”, and “Low-Resource Settings”. Peer-reviewed articles, technical reports, policy documents, and guidance published by international organizations, including the World Health Organization (WHO), were considered where relevant to the review objective. Studies were selected based on their relevance to AI-driven diagnostic systems, explainability techniques, implementation challenges, and healthcare applications within resource-constrained environments.

Literature searches were conducted between January and April 2026 using PubMed, Scopus, Web of Science, IEEE Xplore and Google Scholar. Searches included studies published in English between January 2015 and April 2026 to capture contemporary developments in explainable artificial intelligence and digital diagnostics. Search terms were combined using Boolean operators, including (“Artificial Intelligence” OR “Machine Learning” OR “Deep Learning”) AND (“Explainable Artificial Intelligence” OR “XAI”) AND (“Neglected Tropical Diseases” OR malaria OR schistosomiasis OR leishmaniasis OR leprosy) AND (diagnosis OR medical imaging OR computer vision). A total of 864 records were identified. After removing duplicates (*n* = 173), 691 titles and abstracts were screened. Following relevance assessment, 126 full-text articles were reviewed, from which 82 publications informed the narrative synthesis and framework development. Peer-reviewed original studies, reviews, methodological papers, WHO guidance documents and digital health policy reports relevant to AI-enabled NTD diagnosis were included, whereas editorials lacking methodological insight, conference abstracts without full texts and unrelated AI applications were excluded.

**Figure 1:**
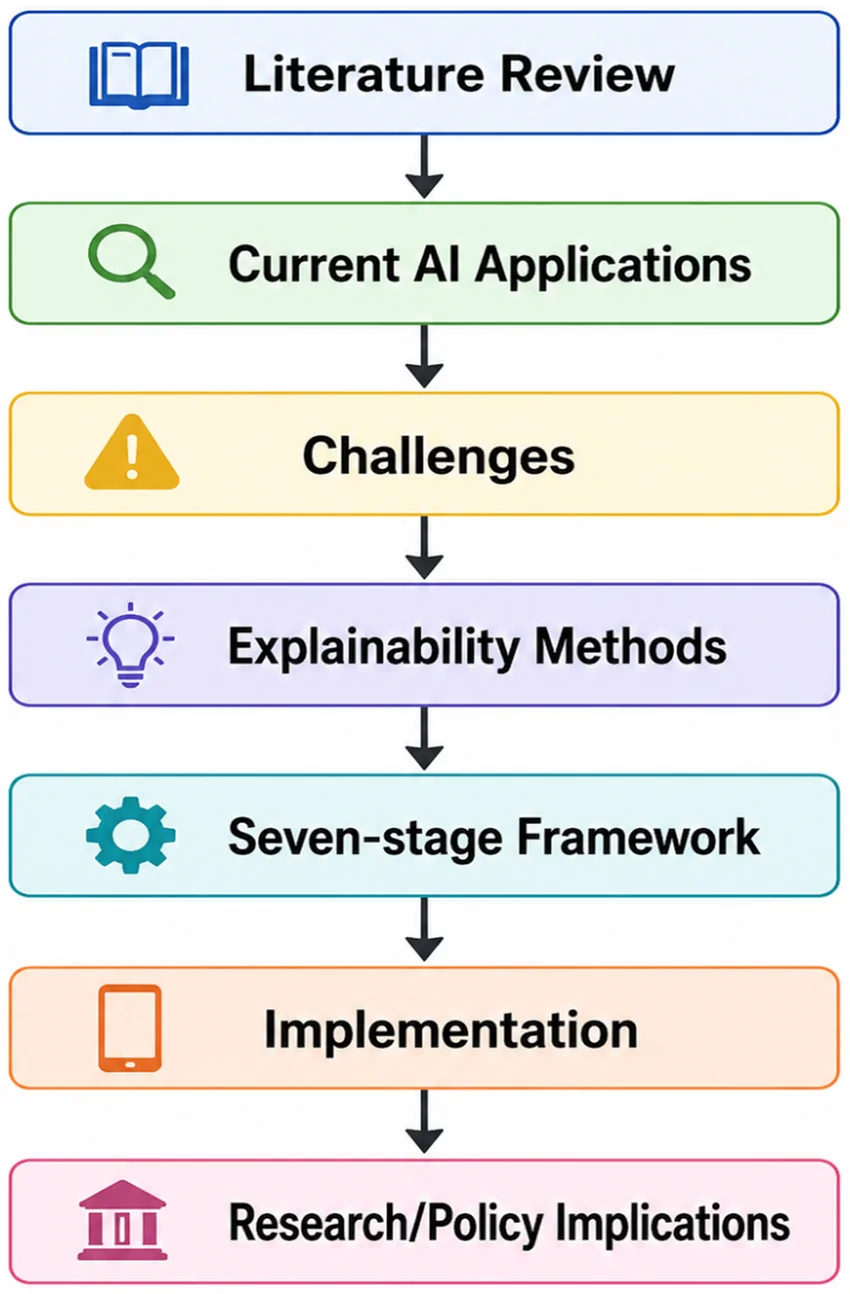
Overall organization of the review. The findings first synthesize the current applications of artificial intelligence (AI) for neglected tropical disease (NTD) diagnosis, critically examines implementation challenges and existing explainability approaches, proposes a seven-stage explainable AI framework, and concludes by discussing implementation strategies together with research, policy, and practice implications.

The proposed framework was developed through thematic synthesis of the identified literature, integrating evidence from AI development studies, explainability research, digital health implementation frameworks, and global health system perspectives. The resulting framework was iteratively refined to address the unique clinical, technical, ethical, and operational challenges associated with deploying explainable AI models for NTD diagnosis in low-resource settings (Figure 1).

### Current applications of artificial intelligence in neglected tropical disease diagnosis

The application of AI to NTD diagnosis has evolved considerably over the past decade, driven by advances in deep learning, computer vision, mobile health technologies, and digital microscopy (Table 1). While AI adoption within mainstream medical specialties such as oncology, radiology, and cardiology has accelerated rapidly, its integration into NTD programmes has been comparatively slower despite the substantial diagnostic challenges that characterize endemic settings. This disparity is particularly noteworthy because many NTDs rely heavily on visual interpretation of clinical manifestations or microscopic findings, making them inherently suitable for image-based machine learning applications. Consequently, a growing body of research has explored how CNNs, object detection algorithms, segmentation models, and hybrid deep learning architectures can augment diagnostic capacity where access to specialist expertise is limited (5, 7, 11).

**Table 1:** Applications of artificial intelligence in neglected tropical disease diagnosis.

Disease	AI Method	Data Type	Reported Use
Malaria	Convolutional Neural Networks (CNNs), Deep Learning	Blood smear microscopy images	Automated parasite detection, species classification, parasite quantification
Schistosomiasis	Deep Learning, Object Detection Models	Urine and stool microscopy images	Egg detection, automated counting, diagnostic support
Soil-Transmitted Helminths	CNNs, Image Recognition Algorithms	Stool microscopy images	Detection and classification of helminth eggs
Cutaneous Leishmaniasis	CNNs, Computer Vision Models	Clinical skin photographs	Lesion classification and disease differentiation
Leprosy	Deep Learning Models	Dermatological images	Skin lesion recognition and screening support
Buruli Ulcer	CNNs and Transfer Learning Models	Skin lesion photographs	Early lesion identification and classification
Yaws	Computer Vision and Deep Learning	Clinical skin images	Automated lesion recognition and differential diagnosis
Onchocerciasis	Machine Learning Algorithms	Microscopy and skin biopsy images	Parasite identification and infection assessment
Lymphatic Filariasis	Deep Learning and Image Analysis	Blood microscopy images	Detection of microfilariae and screening support
Multi-disease NTD Platforms	Hybrid AI Frameworks	Smartphone images and digital microscopy	Integrated point-of-care diagnostic decision support

Among NTDs, malaria remains the most extensively investigated disease in AI-assisted diagnostics. Traditional microscopic examination of blood smears requires highly trained personnel and is susceptible to observer variability, particularly in low-transmission settings where parasitaemia may be low. Recent studies have demonstrated that deep learning models can achieve high levels of sensitivity and specificity in automated parasite detection through the analysis of digital microscopy images, often matching expert microscopists under controlled conditions (16, 17). Beyond simple classification, contemporary AI systems are increasingly capable of parasite quantification, species differentiation, and automated quality control, thereby extending their utility from diagnostic support to broader laboratory workflow optimization. Importantly, several smartphone-integrated diagnostic platforms have demonstrated the feasibility of deploying such systems within resource-constrained environments, highlighting the potential for decentralized diagnosis and improved case management (9).

Similarly, significant progress has been reported in the diagnosis of schistosomiasis and soil-transmitted helminth (STH) infections through AI-enabled microscopy. Conventional diagnosis relies on labor-intensive stool or urine examination techniques that are highly dependent on technician expertise and sample quality. Deep learning-based image recognition systems have increasingly been applied to identify and quantify helminth eggs from digitized microscopic images, offering opportunities to improve consistency and scalability of diagnostic services (18, 19). Although several studies have reported promising diagnostic accuracy, challenges remain regarding model generalisability across varying microscope types, image acquisition protocols, and endemic settings. This highlights an important limitation within current AI research, namely that high performance under experimental conditions does not necessarily translate into robust performance in real-world public health programmes.

AI applications have also demonstrated considerable promise in the diagnosis of skin-related NTDs, including cutaneous leishmaniasis, Buruli ulcer, leprosy, and yaws. These diseases frequently present with visible dermatological manifestations that are amenable to computer vision analysis. Deep convolutional neural networks trained on clinical photographs have shown the ability to distinguish between disease-specific lesion patterns and differentiate NTD-related skin conditions from other dermatological disorders with encouraging accuracy (20, 21). Nevertheless, the evidence also reveals important concerns regarding dataset representativeness, particularly the underrepresentation of diverse skin tones that characterize many endemic populations. Failure to address such biases may inadvertently reinforce diagnostic inequities and undermine the reliability of AI systems when deployed in the communities they are intended to serve.

Leishmaniasis represents another emerging area of AI innovation and diagnostic approaches traditionally rely on microscopy, molecular testing, or clinical examination, all of which face accessibility constraints in many endemic regions. Recent machine learning models have been developed to classify cutaneous lesions from digital images and to support parasite identification using microscopic samples, demonstrating encouraging diagnostic performance while reducing reliance on specialist interpretation (22). However, despite these advances, the current literature remains fragmented, often emphasizing predictive accuracy while providing limited insight into model interpretability, clinical integration, or implementation feasibility.

Collectively, these developments suggest that AI possesses substantial potential to transform NTD diagnosis by improving accessibility, consistency, and diagnostic efficiency across resource-limited settings. Yet, a critical observation emerging from the literature is that most studies remain focused on algorithmic performance metrics, with comparatively little attention devoted to explainability, transparency, and end-user trust. As AI systems move closer to clinical deployment, future innovation must therefore extend beyond accuracy optimization towards the development of interpretable diagnostic tools capable of supporting both frontline healthcare workers and public health decision-makers in complex real-world environments (Table 1).

### Why explainability matters in low-resource settings

The growing enthusiasm surrounding AI in global health has largely been driven by remarkable improvements in predictive performance; however, evidence increasingly suggests that accuracy alone is insufficient to guarantee successful implementation within real-world healthcare systems, particularly in low-resource settings where diagnostic decisions often occur under conditions of uncertainty, limited infrastructure, and constrained specialist support (23, 24). In such environments, trust represents a fundamental prerequisite for technology adoption rather than a secondary consideration. Frontline healthcare workers are more likely to engage with diagnostic systems when they can understand the rationale underlying algorithmic recommendations, especially when managing diseases associated with significant morbidity, stigma, and public health consequences. Studies examining clinician attitudes toward AI consistently demonstrate that opaque “black-box” systems generate skepticism, reduce confidence in recommendations, and ultimately hinder integration into clinical workflows, irrespective of their reported performance metrics (25, 26). Consequently, explainability should not be viewed merely as a technical enhancement but rather as a mechanism for establishing cognitive trust between human decision-makers and machine-generated outputs (Table 2).

**Table 2:** Why explainability is essential for AI-enabled NTD diagnosis in low-resource settings.

Domain	Importance of Explainability	Implications for NTD Diagnosis
Trust	Enables users to understand the reasoning behind predictions	Improves confidence among frontline healthcare workers and laboratory personnel
Clinical Adoption	Supports integration of AI outputs into clinical decision-making processes	Enhances acceptance and routine use in endemic settings
Safety	Allows identification of implausible or potentially erroneous predictions	Reduces automation bias and diagnostic errors
Accountability	Facilitates auditing, validation, and regulatory oversight	Strengthens governance and ethical compliance
Health Equity	Reveals potential biases affecting vulnerable populations	Supports fair and equitable diagnostic performance across diverse communities
Human–AI Collaboration	Promotes shared decision-making between clinicians and AI systems	Improves diagnostic accuracy while preserving human oversight
Public Trust	Increases transparency of AI-enabled healthcare interventions	Encourages community acceptance of digital health innovations
Scalability	Supports deployment across different health systems and settings	Enhances sustainability and implementation success in LMICs

Beyond trust, explainability plays a critical role in facilitating meaningful clinical adoption. Diagnostic decisions in NTD-endemic regions are frequently made by community health workers, laboratory technicians, and non-specialist clinicians who must balance algorithmic recommendations against contextual clinical knowledge. An explainable model that identifies the specific lesion characteristics, parasite structures, or image regions contributing to a prediction can support clinical reasoning and improve user confidence in decision-making processes (27). By contrast, a highly accurate but uninterpretable system may paradoxically increase uncertainty because healthcare workers cannot determine whether predictions are clinically plausible or potentially erroneous. This distinction becomes particularly important in settings where opportunities for expert consultation are limited and diagnostic errors may remain unchallenged for prolonged periods.

Safety considerations further strengthen the case for explainability in AI-enabled NTD diagnosis. While machine learning models may achieve impressive performance during development, their reliability can deteriorate when exposed to new populations, different imaging devices, variable environmental conditions, or previously unseen disease presentations. Explainable systems provide an additional layer of safety by enabling users to identify situations in which model reasoning appears inconsistent with clinical expectations, thereby supporting appropriate human oversight and reducing the risk of automation bias (28). In this regard, explainability functions as an important safeguard against inappropriate reliance on algorithmic outputs, particularly in resource-constrained settings where formal regulatory mechanisms for AI oversight may still be evolving.

The importance of accountability has likewise become increasingly prominent within discussions surrounding healthcare AI governance. Unlike conventional software, machine learning systems continuously learn from data and may evolve over time, creating challenges regarding responsibility for erroneous predictions and adverse outcomes. Transparent models facilitate auditability by allowing researchers, healthcare institutions, regulators, and policymakers to evaluate how diagnostic conclusions are generated, thereby strengthening governance structures and supporting compliance with emerging ethical and regulatory frameworks (24, 29). Without explainability, meaningful accountability becomes difficult because stakeholders lack the necessary visibility to assess whether decisions are clinically justified, technically robust, or influenced by hidden biases.

Perhaps most importantly, explainability is intrinsically linked to health equity, a principle that lies at the heart of global NTD elimination strategies. Numerous studies have demonstrated that AI systems can inherit biases from training datasets, potentially resulting in differential performance across populations defined by geography, ethnicity, skin pigmentation, socioeconomic status, or healthcare access (30, 31). Such disparities are particularly concerning for NTDs, which disproportionately affect historically marginalized populations that are often underrepresented in digital health datasets. Explainable AI offers a pathway for identifying and mitigating these biases by revealing the features and patterns driving algorithmic decisions, thereby enabling continuous evaluation of fairness across diverse populations. Consequently, explainability should be regarded not only as a technological objective but also as an ethical imperative for ensuring that AI-driven innovations contribute to reducing, rather than exacerbating, existing global health inequalities.

### Existing explainability techniques relevant to NTD diagnosis

While high-performing models may accurately classify lesions, detect parasites, or identify disease-specific patterns, healthcare professionals often remain unable to understand the reasoning processes underlying these predictions. This challenge is particularly significant in NTD-endemic settings where diagnostic decisions frequently carry substantial clinical and public health consequences and where specialist expertise may be unavailable to independently verify algorithmic outputs. Consequently, explainability techniques have emerged as a critical component of trustworthy artificial intelligence (AI), providing mechanisms through which users can visualize, interpret, and evaluate model decision-making processes rather than relying solely on predictive outputs (23, 27).

Among the most widely adopted approaches in medical imaging is Gradient-weighted Class Activation Mapping (Grad-CAM), which generates visual explanations by highlighting image regions that contribute most strongly to a model's prediction. In the context of NTD diagnosis, Grad-CAM can identify lesion boundaries in cutaneous leishmaniasis, focal skin abnormalities in leprosy, or parasite-containing regions within microscopic images, thereby enabling clinicians to assess whether the model is focusing on biologically relevant features (32). The intuitive visual nature of Grad-CAM has contributed significantly to its popularity within healthcare AI, particularly because it provides explanations that are accessible even to users without advanced computational expertise. Closely related heatmap-based approaches similarly offer spatial visualizations of model attention, allowing healthcare workers to examine the anatomical or microscopic structures influencing diagnostic predictions. Although such techniques enhance transparency, critics argue that heatmaps may occasionally oversimplify complex model reasoning and should therefore be interpreted cautiously rather than regarded as definitive explanations (28).

Beyond image localization methods, model-agnostic approaches such as Local Interpretable Model-Agnostic Explanations (LIME) and Shapley Additive Explanations (SHAP) have gained prominence due to their ability to explain predictions across diverse machine learning architectures. LIME generates localized surrogate models that approximate decision-making behaviour around individual predictions, thereby enabling users to identify the features most responsible for a specific diagnostic output (33). Similarly, SHAP applies principles derived from cooperative game theory to quantify the contribution of individual variables toward model predictions, producing both local and global explanations that support comprehensive model evaluation (34). Although these approaches offer substantial interpretive value, their computational complexity and sensitivity to input perturbations may present practical challenges for deployment in resource-constrained settings where computational infrastructure is limited (Table 3).

**Table 3:** Explainability approaches for medical imaging AI and their relevance to NTD diagnosis.

Method	Description	Advantages	Limitations	Suitability for NTD Diagnosis
Grad-CAM	Generates visual maps highlighting image regions contributing to predictions	Intuitive visualization, easy clinician interpretation, widely adopted in medical imaging	Limited granularity, may not fully capture model reasoning	High
Heatmaps	Visual representation of areas receiving greater model attention	Easy to understand, useful for lesion and parasite localization	Risk of oversimplification, potential misinterpretation	High
SHAP (Shapley Additive Explanations)	Quantifies feature contributions using game-theoretic principles	Strong theoretical foundation, provides local and global explanations	Computationally intensive, resource demanding	Moderate
LIME (Local Interpretable Model-Agnostic Explanations)	Generates local surrogate models around individual predictions	Model-agnostic, useful for case-specific explanations	May produce unstable explanations across repeated analyses	Moderate
Attention Mechanisms	Highlights features prioritized during model learning and prediction	Built directly into model architecture, supports intrinsic interpretability	Debate regarding explanatory validity, architecture-dependent	High
Saliency Maps	Measures sensitivity of predictions to image pixels	Simple implementation, rapid visualization	Can be noisy and difficult to interpret clinically	Moderate
Explainable Segmentation Models	Delineates disease-specific structures while providing classification outputs	Supports clinical validation and lesion localization	Requires high-quality annotated datasets	High
Hybrid Explainability Frameworks	Combines multiple explanation methods within a single system	More comprehensive interpretation and robustness	Increased computational complexity	Very High

More recently, attention mechanisms have emerged as an intrinsic explainability strategy within deep learning architectures. Unlike *post-hoc* methods that attempt to explain decisions after model training, attention mechanisms enable networks to explicitly prioritize informative regions or features during prediction, thereby providing insights into how information is processed throughout the diagnostic workflow (35). This capability may be particularly valuable for NTD diagnosis because disease manifestations are often subtle, heterogeneous, and influenced by environmental factors. By revealing which image regions or feature representations receive the greatest analytical emphasis, attention-based models offer a promising pathway toward integrating explainability directly into model design rather than treating it as an external add-on. Nevertheless, despite growing enthusiasm, substantial debate remains regarding whether attention weights alone constitute true explanations, highlighting the need for careful evaluation of explainability methods before widespread implementation in clinical practice (36).

### Challenges to developing explainable AI for NTD diagnosis

Despite growing optimism regarding the potential of XAI to transform NTD diagnosis, substantial challenges continue to constrain the development, validation, and implementation of robust systems suitable for low-resource settings. Perhaps the most fundamental obstacle relates to data availability. Unlike diseases that benefit from extensive digitized clinical repositories, many NTDs remain underrepresented in global health datasets due to chronic underfunding, fragmented surveillance systems, and limited research infrastructure. Consequently, AI developers frequently rely on relatively small datasets that may not adequately capture the biological, demographic, and geographical diversity of affected populations. Small datasets increase the risk of overfitting, whereby models learn dataset-specific characteristics rather than clinically meaningful patterns, resulting in inflated performance during development but poor performance when exposed to real-world populations (37, 38). Equally problematic is the issue of label quality. The effectiveness of supervised machine learning depends heavily on accurate annotations; however, many NTD datasets are labelled using routine clinical diagnoses or microscopy results that may themselves be subject to human error and inter-observer variability. Inconsistent annotation practices can therefore propagate uncertainty throughout the model development pipeline, ultimately affecting both predictive performance and explainability outputs (39).

Beyond data limitations, technical challenges remain significant. A persistent concern within medical AI research is the lack of generalisability across different populations and settings. Models trained using data from a particular country, healthcare facility, imaging device, or patient population frequently experience performance degradation when applied elsewhere, reflecting differences in disease presentation, environmental conditions, diagnostic workflows, and population characteristics (40). This challenge is especially relevant for NTDs, whose epidemiological patterns vary considerably across endemic regions. Furthermore, explainable AI systems are not immune to model drift, a phenomenon whereby predictive performance gradually deteriorates over time as underlying data distributions change. Factors such as evolving disease epidemiology, changing imaging technologies, or shifts in healthcare practices may alter the characteristics of incoming data, rendering previously reliable models less accurate and potentially generating misleading explanations if continuous monitoring mechanisms are absent (41).

The successful deployment of explainable AI in NTD programmes is further complicated by health-system constraints that characterize many endemic regions. Although AI technologies are frequently promoted as solutions for resource-limited environments, their implementation often assumes the availability of digital infrastructure that may not exist in practice. In many low- and middle-income countries (LMICs), healthcare facilities continue to face unreliable electricity supplies, inadequate computing resources, insufficient maintenance capacity, and shortages of trained personnel capable of managing digital technologies (42). Internet connectivity presents an additional challenge. While cloud-based AI systems can support sophisticated analytics and continuous model updates, many rural communities experience intermittent or absent internet access, limiting the feasibility of real-time data transfer and remote computational processing (43). Consequently, explainable AI systems intended for NTD diagnosis must be designed with offline functionality and computational efficiency in mind rather than assuming constant digital connectivity.

Ethical considerations present equally complex barriers to implementation. Algorithmic bias remains one of the most widely discussed concerns in healthcare AI because models trained on unrepresentative datasets may systematically underperform among marginalized populations, thereby reinforcing existing health inequalities rather than alleviating them (44). This issue is particularly relevant to NTDs because affected populations are frequently underrepresented in global digital health datasets despite bearing the highest disease burden. Moreover, explainability itself does not automatically guarantee fairness or trustworthiness. Some explanation methods may create an illusion of transparency without accurately reflecting underlying decision-making processes, potentially misleading users into overestimating system reliability (45). As a result, transparency should be viewed as a necessary but insufficient condition for ethical AI deployment. Effective implementation will require a broader governance framework encompassing fairness assessment, accountability mechanisms, stakeholder engagement, and continuous performance monitoring to ensure that explainable AI systems contribute meaningfully to equitable and trustworthy NTD diagnosis.

### Proposed framework for developing explainable AI models for NTD diagnosis

**Figure 2:**
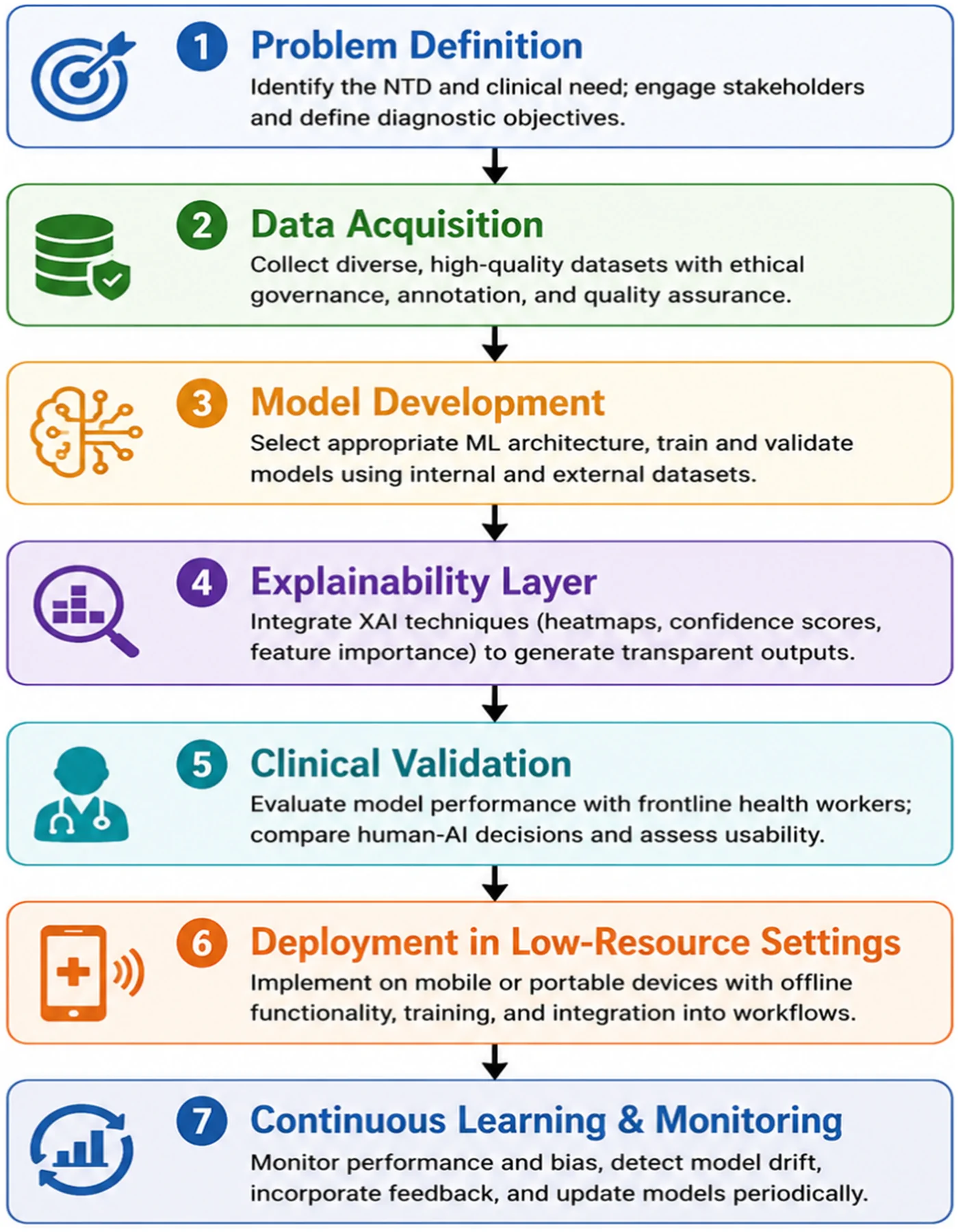
Proposed seven-stage framework for developing explainable artificial intelligence models for neglected tropical disease diagnosis in low-resource settings. The framework illustrates a sequential and iterative development pathway comprising problem definition, data acquisition, model development, explainability layer integration, clinical validation, deployment in low-resource settings, and continuous learning and monitoring. By embedding explainability throughout the model development lifecycle rather than treating it as a *post hoc* addition, the framework aims to improve transparency, trustworthiness, clinical adoption, and sustainable implementation of AI-enabled diagnostic systems in resource-constrained environments.

To address this gap, we propose a seven-stage framework that integrates explainability throughout the AI development lifecycle rather than treating it as a post-development addition (Table 4). The framework is designed to support the creation of clinically meaningful, ethically responsible, and operationally feasible diagnostic systems capable of serving endemic communities while maintaining transparency and trust (Figure 2).

**Table 4:** Proposed explainable AI framework for NTD diagnosis in low-resource settings.

Stage	Objective	Key Activities	Expected Output
1. Problem Definition	Identify the diagnostic challenge and public health priority	Disease selection, stakeholder engagement, clinical need assessment	Clearly defined clinical problem and implementation goal
2. Data Acquisition	Develop high-quality and representative datasets	Dataset collection, annotation, quality assurance, ethical governance	Diverse and ethically governed diagnostic dataset
3. Model Development	Build and validate diagnostic algorithms	Architecture selection, training, testing, internal and external validation	Robust diagnostic AI model
4. Explainability Layer Integration	Enhance transparency and interpretability	Grad-CAM, heatmaps, confidence scores, SHAP, attention mechanisms	Transparent and interpretable predictions
5. Clinical Validation	Assess real-world clinical utility and trustworthiness	Human–AI comparison, usability testing, frontline worker evaluation	Evidence of clinical acceptability and trust
6. Deployment in Low-Resource Settings	Ensure operational feasibility and scalability	Mobile optimization, offline functionality, user training, governance planning	Sustainable real-world implementation
7. Continuous Learning and Monitoring	Maintain performance, fairness, and accountability	Bias monitoring, model drift detection, feedback systems, periodic updating	Continuous model improvement and long-term reliability

The first stage, Problem Definition, focuses on identifying the specific NTD and clinical challenge that the AI system seeks to address. Disease selection should be guided by public health priorities, diagnostic bottlenecks, disease burden, and implementation feasibility within endemic settings. Importantly, clinical stakeholders, including frontline healthcare workers, laboratory personnel, public health practitioners, and policymakers, should be involved from the outset to ensure that model objectives align with genuine healthcare needs rather than purely technological opportunities (24). Such early stakeholder engagement strengthens contextual relevance and reduces the risk of developing technically sophisticated solutions that ultimately fail to address real-world diagnostic challenges.

The second stage, Data Acquisition, involves the development of diverse, representative, and ethically governed datasets. Given the considerable heterogeneity of NTD manifestations across geographical regions, demographic groups, imaging devices, and healthcare settings, datasets should intentionally incorporate variation to enhance model robustness and fairness. Ethical governance mechanisms including informed consent, data anonymization, privacy protection, and transparent data-sharing agreements must be established to ensure responsible use of health information and maintain public trust (46).

The third stage, Model Development, encompasses the selection of appropriate machine learning architectures, including convolutional neural networks, object detection frameworks, segmentation models, or hybrid approaches depending on the diagnostic task. Rigorous internal and external validation should be conducted to evaluate performance across diverse datasets and operational environments. Importantly, model success should not be assessed solely through conventional metrics such as accuracy, sensitivity, and specificity but also through measures of robustness, calibration, and generalisability (47).

The fourth stage, Explainability Layer Integration, represents the central innovation of the proposed framework. Rather than developing black-box models and subsequently attempting to explain them, explainability should be embedded directly within system design. Techniques such as Grad-CAM heatmaps, attention visualization, confidence scoring, feature attribution methods, and uncertainty estimation should be incorporated to provide users with transparent insights into model reasoning. This layer transforms AI outputs from simple predictions into interpretable decision-support tools capable of supporting human judgement and fostering trust among end-users (23).

The fifth stage, Clinical Validation, recognizes that technical performance alone does not guarantee clinical utility. Models should therefore undergo evaluation by frontline healthcare workers and domain experts operating within intended deployment environments. Human–AI comparison studies, usability assessments, and workflow integration testing can provide valuable evidence regarding interpretability, trustworthiness, and practical effectiveness. Such evaluations are particularly important in NTD-endemic settings where healthcare workers may possess varying levels of technical expertise and where diagnostic support systems must complement rather than disrupt existing clinical workflows (25).

The sixth stage, Deployment in Low-Resource Settings, focuses on operational implementation. To maximize accessibility and scalability, AI systems should be optimized for mobile devices, portable diagnostic platforms, and offline functionality. Dependence on continuous internet connectivity should be minimized because many endemic communities continue to experience substantial digital infrastructure constraints. Additionally, deployment strategies should incorporate user training, technical support mechanisms, and clear governance structures to facilitate sustainable adoption (43). Financial sustainability should also be considered during implementation. The framework intentionally prioritizes deployment through smartphones, low-cost digital microscopes and edge-computing architectures that minimise dependence on expensive cloud infrastructure. Open-source machine learning libraries, offline inference and integration into existing primary healthcare systems may substantially reduce implementation costs. Nevertheless, successful deployment will require investment in workforce training, device maintenance and periodic software updates, which should be incorporated into national digital health budgets and donor-supported NTD programmes.

Finally, the seventh stage, Continuous Learning and Monitoring, acknowledges that AI systems exist within dynamic healthcare environments. Post-deployment monitoring should assess model performance, identify emerging biases, detect model drift, and evaluate fairness across different population groups. Regular model updating, transparency reporting, and stakeholder feedback mechanisms should be incorporated to ensure ongoing reliability and accountability. By embedding continuous evaluation within the framework, explainable AI systems can evolve responsibly while maintaining clinical relevance and public trust over time (29). Although the proposed framework has been developed through comprehensive synthesis of current evidence, it remains conceptual and requires empirical validation. Future studies should prospectively evaluate the framework using pilot implementations across multiple NTD-endemic settings, comparing diagnostic performance, clinician trust, usability, implementation outcomes and patient-level impact. Such validation studies will provide important evidence regarding the framework's effectiveness, scalability and generalisability.

### Comparison of the proposed framework with existing AI development frameworks

Unlike existing AI development frameworks that primarily emphasize algorithm development, governance or regulatory oversight, the proposed framework explicitly integrates explainability throughout the model lifecycle while simultaneously addressing the unique clinical, infrastructural and implementation challenges associated with neglected tropical disease diagnosis in resource-constrained settings. This combination distinguishes the framework from existing approaches and positions it as a practical roadmap for translational AI in global health (Table 5).

**Table 5:** Comparison of the proposed framework with existing AI development frameworks.

Framework	Explainability	Clinical Validation	Low-resource focus	Continuous Monitoring	NTD-specific
CRISP-DM	Limited	No	No	No	No
WHO AI Ethics Framework	General principles	Partial	Partial	Yes	No
FDA Good Machine Learning Practice	Partial	Yes	No	Yes	No
Existing Medical Imaging AI Pipelines	Usually post-hoc	Variable	No	Rare	No
Proposed Framework	Embedded throughout development	Yes	Yes	Yes	Yes

### Implications for research, policy, and practice

The proposed framework has important implications for future research, policy development, and clinical practice, particularly as global health systems increasingly explore AI as a tool for accelerating progress toward NTD control and elimination targets. From a research perspective, there remains a substantial need for NTD-specific XAI studies that move beyond reporting predictive performance metrics to evaluate transparency, trustworthiness, fairness, and real-world usability. Current evidence remains heavily concentrated on algorithm development, while comparatively little attention has been devoted to understanding how healthcare workers interpret AI-generated explanations or how explainability influences diagnostic decision-making in endemic settings. Furthermore, the absence of standardized benchmark datasets continues to hinder meaningful comparison between competing models and limits external validation efforts. Establishing large, diverse, and openly accessible NTD image repositories will therefore be essential for advancing reproducible and equitable AI innovation (24, 37).

From a policy perspective, explainable AI aligns closely with emerging global priorities surrounding digital health governance. The World Health Organization has repeatedly emphasized that AI technologies should be transparent, accountable, and ethically deployed to ensure that digital innovation contributes positively to health outcomes while protecting vulnerable populations (24). Consequently, national governments, regulatory agencies, and development partners should prioritize governance frameworks that mandate explainability, fairness assessment, data protection, and continuous monitoring for AI-enabled diagnostic systems. Such policies will be particularly important in low- and middle-income countries where regulatory structures for healthcare AI remain underdeveloped.

The practical implications are equally significant. Explainable diagnostic systems have the potential to enhance decision-making among frontline healthcare workers by providing interpretable diagnostic support in settings where specialist expertise is scarce. Through combining mobile technologies, offline functionality, and transparent decision-support mechanisms, platforms illustrate how explainable AI can bridge diagnostic gaps in rural and underserved communities while simultaneously generating valuable epidemiological insights for disease surveillance and health system strengthening. Ultimately, the integration of explainability into AI-enabled diagnostics may facilitate greater trust, wider adoption, and more sustainable deployment of digital health innovations across NTD-endemic regions.

## Conclusion

Artificial intelligence is increasingly recognized as a transformative technology capable of addressing longstanding diagnostic challenges associated with NTDs, particularly in low-resource settings where shortages of specialist expertise, laboratory infrastructure, and diagnostic capacity continue to hinder progress toward disease control and elimination targets. However, the evidence reviewed in this paper demonstrates that the future success of AI-enabled NTD diagnostics will depend not solely on predictive accuracy but on the ability of these systems to generate transparent, interpretable, and trustworthy outputs that can be confidently integrated into routine healthcare practice. While advances in deep learning, computer vision, and mobile diagnostics have shown considerable promise across diseases such as malaria, schistosomiasis, leishmaniasis, and other NTDs, significant challenges remain regarding explainability, bias, generalisability, accountability, and real-world implementation.

To address these challenges, this paper proposes a structured seven-stage framework that embeds explainability throughout the AI development lifecycle, from problem definition and data acquisition to deployment and continuous monitoring. By integrating explainability mechanisms alongside technical development, clinical validation, and health-system considerations, the framework seeks to bridge the gap between algorithmic innovation and practical healthcare application. Importantly, it positions trust, transparency, equity, and usability as fundamental design principles rather than secondary considerations. As global efforts to strengthen digital health systems continue to expand, the adoption of explainable AI frameworks may provide a sustainable pathway for developing diagnostic tools that are not only technically robust but also clinically meaningful, ethically responsible, and implementable within the resource-constrained environments where NTDs remain most prevalent. Future work will focus on implementing and validating the proposed framework through the development of explainable AI diagnostic platforms for skin and microscopy-based neglected tropical diseases. Such implementation studies will generate empirical evidence regarding usability, diagnostic performance, cost-effectiveness and scalability within real-world healthcare systems.

## Data Availability

The original contributions presented in the study are included in the article/Supplementary Material, further inquiries can be directed to the corresponding author.
